# Predictors of Acute Encephalopathy in Patients with COVID-19

**DOI:** 10.3390/jcm10214821

**Published:** 2021-10-20

**Authors:** Oleg I. Vinogradov, Tatyana K. Ogarkova, Kamila V. Shamtieva, Pavel V. Alexandrov, Astanda V. Mushba, Daria S. Kanshina, Daria V. Yakovleva, Maria A. Surma, Ilia S. Nikolaev, Nadezhda Kh. Gorst

**Affiliations:** Federal State Budgetary Institution “National Medical and Surgical Center Named after N.I. Pirogov”, Ministry of Healthcare of the Russian Federation, 105203 Moscow, Russia; olvinog@mail.ru (O.I.V.); kamila.shamt@gmail.com (K.V.S.); alexandrov-pavelmd@yandex.ru (P.V.A.); astanda.mushba@mail.ru (A.V.M.); dr.d.kanshina@gmail.com (D.S.K.); maria_fnc@mail.ru (M.A.S.); niko.ilya2013@yandex.ru (I.S.N.); GorstHope@gmail.com (N.K.G.)

**Keywords:** COVID-19, SARS-CoV-2, delirium, encephalopathy

## Abstract

Introduction: The majority of patients with severe COVID-19 suffer from delirium as the main sign of encephalopathy associated with this viral infection. The aim of this study was to identify early markers of the development of this condition. Materials: The prospective cohort-based study included patients with community-acquired pneumonia and confirmed pulmonary tissue infiltration based on CT data, with a lesion consisting of at least 25% of one lung. The main group included patients who have developed acute encephalopathy (10 patients, 3 (30%) women; average age, 47.9 ± 7.3 years). The control group included patients who at discharge did not have acute encephalopathy (20 patients, 11 (55%) women; average age, 51.0 ± 10.5 years). The study collected clinical examination data, comprehensive laboratory data, neurophysiological data, pulse oximetry and CT data to identify the predictors of acute encephalopathy (study ClinicalTrials.gov identifier NCT04405544). Results: Data analysis showed a significant relationship between encephalopathy with the degree of lung tissue damage, arterial hypertension, and type 2 diabetes mellitus, as well as with D-dimer, LDH, and lymphopenia. Conclusions: The development of encephalopathy is secondary to the severity of the patient’s condition since a more severe course of the coronavirus infection leads to hypoxic brain damage.

## 1. Introduction

Damage of the central and peripheral nervous system is a common complication of any respiratory infection [1]. The newly emerged coronavirus, like other similar viral agents, can lead to a wide range of neurological complications.

It is estimated that more than one third of patients with COVID-19 develop neurological symptoms, including headaches, paresthesia, impaired consciousness and neuropsychiatric symptoms, which appear to be associated with a more severe course of the disease [2]. It is also known that up to 88% of patients have symptoms such as anosmia or ageusia, which confirms the tropism of the virus with the nervous tissue [3]. Several studies have shown that up to 65% of patients with severe COVID-19 and up to 75% of patients undergoing artificial ventilation in intensive care units suffer from delirium at some point during their hospitalization [4,5,6]. Furthermore, a series of cases from Wuhan showed that at least 20% of patients who eventually died from COVID-19 had had signs of encephalopathy [3,7]. The development of delirium follows the stress vulnerability model with precipitating factors that include severe infection, acute respiratory distress, invasive ventilation in ICU settings, and old age [8].

Clinical manifestations of delirium include disorganized thinking, inattention, euphoria, language impairment, hallucinations, reversal of the sleep-wake cycle, disorientation, and memory deficit [9]. Delirium is the main clinical sign of the type of encephalopathy associated with respiratory infections [10]. This complication is one of the determining factors in the management of patients since the acute violation of higher mental functions obliges a transfer of the patient to an intensive care unit. The occurrence of delirium is an independent predictor of higher mortality, higher treatment costs, and a longer period of treatment for patients in intensive care units.

Alternate expressions of delirium associated with COVID-19 may include hypoxemia and oxidative stress as a result of acute respiratory distress syndrome, as well as hypoperfusion and toxic effects of metabolites accumulated in the body as a result of multiple organ failure [11,12]. As described in other cases, this group of patients has disseminated intravascular coagulation syndrome [3], which can also lead to brain damage through the failure of cerebral microcirculation.

Data on delirium related to SARS-CoV-2 is still very limited. The study of predictors of delirious states, other clinical signs of encephalopathy, and a whole range of other neurological disorders is important in the context of a new coronavirus pandemic. Therefore, we carried out a prospective cohort study to identify predictors of acute encephalopathy in patients with COVID-19.

## 2. Materials and Methods

This prospective cohort-based study on the acute encephalopathy predictors in patients with COVID-19 was conducted at the Pirogov National Medical and Surgical Center (Moscow, Russia) between April and July 2020 (NCT04405544).

The study included patients with community-acquired pneumonia and confirmed pulmonary tissue infiltration based on CT data, with a lesion consisting of at least 25% of one lung and changes corresponding with a high probability of coronavirus pneumonia (CO-RADS 4–5) [13].

In the further course of the disease, the patients were divided into two groups (cohorts). The main group included patients who had developed acute encephalopathy (10 patients, 3 (30%) women; average age, 47.9 ± 7.3 years). The control group included patients who at discharge did not have acute encephalopathy (20 patients, 11 (55%) women; average age, 51.0 ± 10.5 years). We did not divide patients by subtypes of delirium (hyperactive, hypoactive, mixed) due to a limited sample.

The term “acute encephalopathy” refers to a rapidly developing pathobiological process in the brain occurring over less than 4 weeks which leads to a change from the baseline cognitive status to the form of a decreased level of consciousness or delirium [14]. The term “delirium” refers to a clinical state characterized by a combination of features defined by the DSM-5 [15].

### 2.1. Inclusion Criteria

(1) Men and women aged 18–60; (2) community-acquired pneumonia with confirmed pulmonary tissue infiltration according to CT, with changes corresponding to the average- and high-probability indicators of coronavirus pneumonia (levels 4–5 of the CO-RADS classification) and a lesion of more than 25% of one of the lungs; (3) the patient has read the information sheet and signed the informed consent form.

### 2.2. Exclusion Criteria

(1) A negative oropharyngeal swab PCR test for SARS-CoV-2 RNA; (2) history of myocardial infarction or stroke; (3) verified thrombophilia; (4) pregnancy; (5) patients with a history of malignant tumors, including a postoperative period relating to chemotherapy and/or radiation therapy; (6) acute stroke; (7) previous diagnosis of psychiatric/neurological disorders.

### 2.3. Dropout Criteria

The patient’s refusal to participate further in the study.

The study was approved by the Local Ethics Committee of the Pirogov National Medical and Surgical Center (Moscow, Russia), with the ethics statement No. 8 dated 21 May 2020. All the subjects signed an informed consent form for participation in the study and the processing of their personal data.

The criteria of diagnosis and discharge of COVID-19 patients were based on the polymerase chain reaction (PCR) result according to the World Health Organization (WHO) guidelines [16].

The coronavirus infection was confirmed with a set of reagents for detecting the RNA of coronaviruses causing severe respiratory infection, MERS-CoV (Middle East respiratory syndrome-related coronavirus) and SARS-CoV-2 (severe acute respiratory syndrome-related coronavirus), in the biological material by means of a polymerase chain reaction (PCR) test with hybridization fluorescence detection using testing system AmpliSens^®^ Cov-Bat-FL (dated 7 April 2020) provided under a contract with the Central Research Institute of Epidemiology of the Rospotrebnadzor (Moscow, Russia)

The study collected clinical examination data, including neurological symptoms, comprehensive laboratory tests, CT scans, and neurophysiological parameters to identify the predictors of acute encephalopathy.

The CT data were acquired using a Philips Brilliance scanner (Philips, Amsterdam, The Netherlands) with the acquisition of a minimum of 64 slices. The degree of lung involvement was graded using a 5-point scale proposed in Wuhan, China [17].

All the patients were assessed for arterial hypertension, type 2 diabetes mellitus, obesity, chronic renal failure, diseases of peripheral arteries and lungs related to anamnesis; also, body mass index and glomerular filtration rate calculation, measurement of blood pressure, and an assessment of fasting glycemia were performed.

Regarding the blood parameters in the study, the maximum values for the entire period of hospitalization were included.

White blood cells (WBCs), lymphocytes, and platelets were measured with Automated Hematology Analyzer XN-1000 (Sysmex, Kobe, Japan). Ferritin, creatinine, alanine transaminase (ALT), aspartate transaminase (AST), lactate dehydrogenase (LDH), and C-reactive protein (CRP) were measured with an AU480 chemistry analyzer (Beckman Coulter, USA). D-dimer, prothrombin time (PT), and activated partial thromboplastin time (aPTT) were measured using a TA Compact automated coagulation analyzer (Diagnostica Stago, Paris, France).

Neurological status examination was carried out upon admission to hospital, upon transfer to the intensive care unit, and upon discharge, and did not reveal correlations between the main group and the control group.

The neurophysiological data, including electroencephalography and evoked potential data, were collected from 21 patients at the beginning of hospitalization.

Both studies were carried out using a 19-channel electroencephalograph for routine EEG studies Neuron-Spectr-3 (Neurosoft, Ivanovo, Russia) and a 2-channel myograph Neuro-MVP-micro (Neurosoft, Ivanovo, Russia) with an assessment of the mean N20 peak latency and peak-to-peak amplitude of P18/N20 on the right and left sides, as well as of the index of slow-wave activity.

These patients also underwent pulse oximetry using PulseOx 7500 (SPO medical, Kfar Saba, Israel) with an assessment of the mean and minimal peripheral oxygen saturation (SpO_2_) during wakefulness and sleep and the oxygen desaturation index.

Statistical analysis was performed using the IBM SPSS 23.0 (IBM SPSS Statistics, version 23.0, IBM Corp., Armonk, NY, USA) and R 3.4.3 (R Foundation for Statistical Computing, Vienna, Austria) software. The main descriptive statistics for the categorical and ordinal variables were the frequency and proportion (%); mean and standard deviation or the median and interquartile range for the quantitative variables. Two-way versions of the statistical criteria were used in all the cases. The null hypothesis was rejected if *p* < 0.05. The qualitative parameters for grouping variable levels were compared using the χ2 test or Fisher’s exact test. The quantitative parameter values were compared using Student’s t-test or the Mann-Whitney u-test depending on the type of distribution. The normality was examined using the Kolmogorov-Smirnov test, and visually with histograms. For the anamnestic risk factors, odds ratios were calculated. The predictive value of the laboratory tests for encephalopathy development in the patients with coronavirus pneumonia was evaluated using ROC analysis and binary logistic regression. Using the ROC curves for each parameter included calculation of the area under the curve, the ideal cutoff value, and their sensitivity and specificity.

## 3. Results

The studied groups did not differ in terms of the demographic data and comorbid conditions (Table 1).

The calculation of the odds ratio showed that type 2 diabetes mellitus and, to a greater extent, arterial hypertension increase the risk of encephalopathy in patients with coronavirus (OR = 1.417 (0.196; 10.227) and 3.5 (0.692; 17.715), respectively).

The severity of lung damage influenced the incidence of encephalopathy in the patients with coronavirus, while smell and taste disturbances were not predictors of encephalopathy in the patients with coronavirus (Table 2).

The neurological status and clinical examination in the main group and the control group on admission to hospital did not have statistically significant differences.

Of the 21 patients who underwent neurophysiological examination and pulse oximetry upon admission to hospital, only two developed encephalopathy. Only one of them showed an increase in the index of slow-wave activity (up to 50%). These patients did not differ in terms of pulse oximetry from the main group (Table 3).

Correlation analysis did not show significant relationships between the parameters of neurophysiological examination and pulse oximetry (Table 4).

Comparison of laboratory parameters showed a higher prothrombin time, D-dimer, and LDH values and a lower lymphocyte value in the patients with encephalopathy (Table 5).

It should be noted that an increase in prothrombin time in patients with encephalopathy may be caused by high doses of anticoagulants due to their more severe condition.

**Figure 1:**
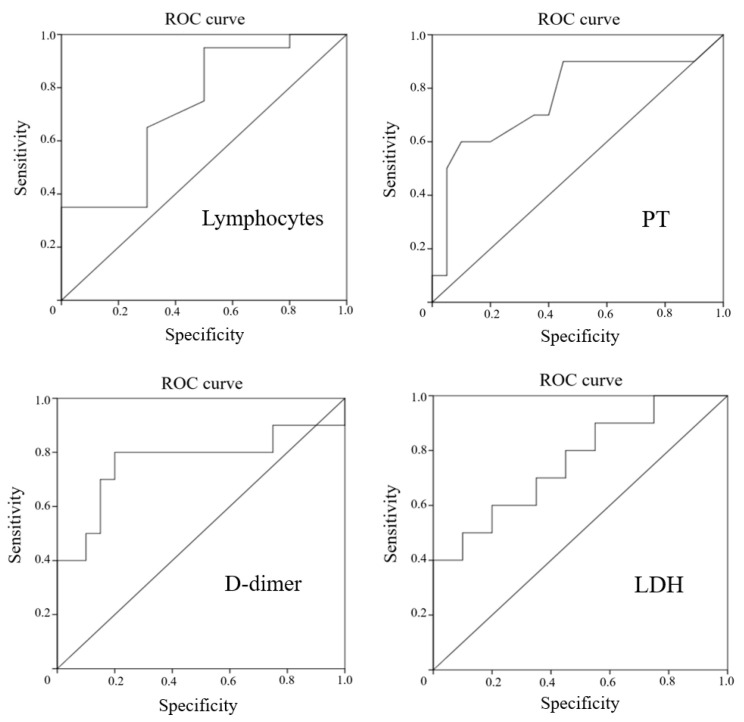
ROC curves for lymphocytes, D-dimer, PT, and LDH in prediction of encephalopathy in patients with COVID-19.

To assess the predictive value of these parameters in order to identify patients with COVID-19 who are at high risk of developing encephalopathy, ROC analysis was performed, which is shown in Figure 1.

All the tests presented had a sufficient area under the curve. Therefore, for all the detected laboratory markers of encephalopathy in the patients with COVID-19, the cutoff values were calculated with the determination of sensitivity and specificity (Table 6).

The optimal values of sensitivity and specificity for the development of encephalopathy in patients with COVID-19 were found for an increase in D-dimer of more than 0.68 μg/mL. A decrease in lymphocytes of less than 0.56 × 10 × 9/L had high sensitivity, but insufficient specificity. Conversely, while the increase in LDH of more than 476.5 U/L and PT of more than 14.4 s were highly specific, the sensitivity remained low.

When applying binary logistic regression to create an equation that would allow the complex use of these indicators, stepwise analysis emphasized only LDH, while D-dimer, PT, and lymphocytes were excluded.

In summary, the most sensitive laboratory markers for the development of encephalopathy in patients with COVID-19 seem to be LDH and D-dimer.

With the development of delirium, treatment was carried out according to the standard protocol.

## 4. Conclusions

According to the current data, the predictors of severe coronavirus pneumonia are age over 65 years old, classic vascular risk factors, immunodeficiency conditions, such laboratory markers as D-dimer, CRP, LDH, troponin, ferritin, CPK, lymphopenia, and some other conditions that have been shown to be associated with the disease in various studies [18,19,20,21,22].

The aim of this study was to identify markers of the development of one of the most common complications of coronavirus pneumonia, namely encephalopathy. The study leveled the effects of age by not including patients over 60 years old. Data analysis showed a significant relationship between encephalopathy with a degree of lung tissue damage, arterial hypertension, and type 2 diabetes mellitus, as well as with D-dimer, LDH, and lymphopenia.

Thus, it can be stated that the development of encephalopathy is secondary to the severity of the patient’s condition since a more severe course of the coronavirus infection leads to hypoxic brain damage with the development of disorders associated with higher cortical functions.

However, it is worth noting that the association of encephalopathy with all the markers of severe coronavirus pneumonia described in the literature is not determined. The most sensitive markers in our study were D-dimer and LDH. For these laboratory markers, earlier studies also described relationships with the development of hypoxic encephalopathy in children [23,24].

Currently, LDH has been shown to be associated with white brain matter damage in patients with a coronavirus infection according to diffusion tensor MRI data [25]. Presumably, LDH, which is one of the key enzymes of the glycolytic pathway and is highly expressed in brain cells, is a sensitive marker of brain tissue damage [26,27].

The association of D-dimer with encephalopathy in patients with a coronavirus infection appears to be related to the damage of the microcirculatory bed of the brain, similar to other organs [28].

In conclusion, we can propose that although encephalopathy is a complication of severe forms of coronavirus pneumonia, not all patients with severe lung damage develop a disorder of higher cortical functions. Thus, patients should be transferred to the intensive care unit even if there are no indications for artificial lung ventilation. This gives rise to the relevance of selecting patients who are most threatened by the development of this complication of the coronavirus infection.

We identified the most sensitive laboratory predictors of encephalopathy in patients with coronavirus pneumonia, which could be determined in the routine practice of any hospital.

## Figures and Tables

**Table 1:** The main demographic data and comorbid conditions in the studied groups.

Parameters	Control Group (*n* = 20)	Main Group (*n* = 10)	*p*-Value
Age, years (mean ± SD)	47.9 ± 7.3	51.0 ± 10.5	0.417
Sex, women (*n*, %)	11 (55%)	3 (30%)	0.260
Arterial hypertension (AH) (*n*, %)	8 (40%)	7 (70%)	0.245
Type 2 diabetes mellitus (DM) (*n*, %)	3 (15%)	2 (20%)	0.999
Obesity (body mass index > 30 kg/m^2^) (*n*, %)	12 (60%)	4 (40%)	0.442
Chronic renal failure (*n*, %)	0 (0%)	0 (0%)	-
Peripheral artery disease (*n*, %)	0 (0%)	0 (0%)	-
Lung disease (*n*, %)	0 (0%)	1 (10%)	0.333

**Table 2:** The clinical features of coronavirus pneumonia in the studied groups.

Parameters	Group without Encephalopathy (*n* = 20)	Group with Encephalopathy (*n* = 10)	*p*-Value
Degree of severity of coronavirus pneumonia (*n*, %)			<0.001
Grade 0–1	0	0	
Grade 2	7 (35%)	0	
Grade 3	11 (55%)	2 (20%)	
Grade 4	2 (10%)	8 (80%)	
Severity of the right lung damage, % (Me (Q25%; Q75%))	50 (40; 75)	75 (75; 80)	0.003
Severity of the left lung damage, % (Me (Q25%; Q75%))	50 (50; 75)	75 (75; 80)	<0.001
Smell disturbances (*n*, %)	7 (35%)	3 (30%)	0.036
Taste disturbances (*n*, %)	4 (20%)	2 (20%)	0.302

**Table 3:** The parameters of pulse oximetry in the patients with and without encephalopathy.

Parameters	Patients without Encephalopathy (*n* = 19)	Patients with Encephalopathy (*n* = 2)	*p*-Value
Mean awake SpO_2_	93.1 (92.6; 94.2)	93.9 (91.4; 96.3)	0.853
Minimal awake SpO_2_	78.5 (73; 84)	76.0 (72; 80)	0.589
Mean asleep SpO_2_	91.8 (86.6; 93.7)	93.2 (90.5; 95.9)	0.589
Minimal asleep SpO_2_	76.5 (71; 82)	84 (79; 89)	0.263
Oxygen desaturation index	23.4 (9.3; 27.1)	4.65 (1.5; 7.8)	0.095

**Table 4:** Parameters of neurophysiological examination and pulse oximetry.

Parameters	Index of Slow-Wave Activity	Mean N20 Peak Latency, Right	Mean N20 Peak Latency, Left	Mean Peak-to-Peak Amplitude of P14/N20, Right	Mean Peak-to-Peak Amplitude of P14/N20, Left
Mean awake SpO_2_	−0.079	−0.201	−0.225	0.015	0.132
Minimal awake SpO_2_	0.175	0.091	0.094	0.182	0.007
Mean asleep SpO_2_	0.088	−0.146	−0.155	−0.509	−0.161
Minimal asleep SpO_2_	0.170	−0.332	−0.371	−0.027	0.101
Oxygen desaturation index	−0.256	−0.230	−0.122	0.347	0.267

**Table 5:** Comparison of laboratory parameters in the studied groups.

Parameters	Group without Encephalopathy (*n* = 20)	Group with Encephalopathy (*n* = 10)	*p*-Value
White blood cells, WBCs (×10^9^/L)	6 (4; 6)	7 (5; 10)	0.120
Lymphocytes (×10^9^/L)	1.07 (0.82; 1.44)	0.69 (0.44; 1.20)	0.044
Platelets (×10^9^/L)	195 (158; 250)	229 (187; 271)	0.142
Alanine transaminase, ALT, U/L	38 (24; 57)	27 (20; 40)	0.267
Aspartate transaminase, AST, U/L	38 (31; 50)	48 (30; 53)	0.475
Lactate dehydrogenase, LDH, U/L	310 (237; 445)	521 (347; 751)	0.022
Creatinine, μmol/L	98 (81; 105)	76 (71; 89)	0.149
C-reactive protein, CRP, μg/L	81 (39; 113)	171 (60; 277)	0.155
Ferritin, μg/L	589 (181; 669)	605 (357; 668)	0.681
D-dimer, μg/mL	0.55 (0.38; 0.65)	1.16 (0.70; 2.53)	0.019
Prothrombin time, PT, s	14 (13; 14)	15 (14; 15)	0.031
Activated partial thromboplastin time, aPTT, s	34 (32; 38)	37 (31; 44)	0.530

**Table 6:** Characteristics of the area under the curve and the cutoff values for the studied markers.

Predictors	AUC	95% CI, Boundary		**Cutoff Values**	Sensitivity	Specificity
		Lower	Upper			
Lactate dehydrogenase, LDH	0.760	0.573	0.947	476.5	60%	80%
D-dimer	0.765	0.547	0.983	0.68	80%	80%
Prothrombin time, PT	0.765	0.565	0.965	14.45	60%	90%
Lymphocytes	0.730	0.531	0.929	0.56	95%	50%

## Data Availability

The data presented in this study are available on request from the corresponding author.

## Abbreviations

The following abbreviations are used in this manuscript:

ALT	Alanine transaminase
aPTT	Activated partial thromboplastin time
ARDS	Acute respiratory distress syndrome
AST	Aspartate transaminase
CPK	Creatine phosphokinase
CRP	C-reactive protein
CT	Computed tomography
DIC	Disseminated intravascular coagulation
DSM-5	Diagnostic and Statistical Manual of Mental Disorders, Fifth Edition
LDH	Lactate dehydrogenase
MERS-CoV	Middle East respiratory syndrome-related coronavirus
PCR	Polymerase chain reaction
PT	Prothrombin time
RNA	Ribonucleic acid
ROC	Receiver operating characteristic
SARS-CoV	Severe acute respiratory syndrome-related coronavirus
WHO	World Health Organization
WBCs	White blood cells
